# *In vitro* exploration of drug-induced thrombotic microangiopathies: clues of diverse endothelial activation pathways respective to interferon-β1a, ciclosporin A, and gemcitabine exposure

**DOI:** 10.3389/fphar.2025.1719192

**Published:** 2026-02-02

**Authors:** Edouard Cubilier, Maxime Taghavi, Eric De Prez, Lucas Jacobs, Sébastien Sinaeve, Joëlle Nortier, Marie-Hélène Antoine

**Affiliations:** 1 Nephrology Department, Brugmann University Hospital, Brussels, Belgium; 2 Laboratory of Experimental Nephrology, Biochemistry Department, Faculty of Medicine, Université Libre de Bruxelles, Brussels, Belgium

**Keywords:** Drug-induced thrombotic microangiopathies, ciclosporin A, gemcitabine, interferon-β1a, complement system, C5b-9, *in vitro*, endothelial cells

## Abstract

Pro-inflammatory and pro-thrombotic stimuli can activate endothelial cells (ECs) and predispose them to thrombotic microangiopathies (TMAs). Drug-induced TMA (DITMA) may occur in clinical practice during treatment with interferon-β1a (IFN-β1a), ciclosporin A (CsA), and gemcitabine (GEM). DITMA may also trigger the complement system and induce membrane attack complex (MAC, C5b-9) deposition *in vivo*, although their role and the benefit of inhibition remain unclear. In an experimental *in vitro* model of microvascular ECs exposed to these three drugs, we searched for MAC deposits and drug-specific pro-inflammatory and pro-thrombotic traits to gain insights into the mechanisms potentially involved in DITMA. Human microvascular endothelial cells line-1 (HMEC-1) was treated with 10% normal human serum, CsA, GEM, and IFN-β1a. Cell viability for each drug was measured using the resazurin assay. Cell component expression of the following markers involved in endothelial pathogenic activation was measured via immunofluorescence and flow cytometry: C5b-9, interleukin (IL)-1α, IL-6, E-selectin, platelet EC adhesion molecule-1 (PECAM-1), intercellular adhesion molecule-1 (ICAM-1), and von Willebrand factor (vWF). Levels of plasminogen activator inhibitor-1 (PAI-1) and urokinase plasminogen activator (uPA) were measured in the supernatants using the enzyme-linked immunosorbent assay (ELISA). Significantly increased C5b-9 deposits were found with each drug, and increased drug-specific activation marker expressions appeared in HMEC-1s when exposed to CsA (IL-1α, IL-6, ICAM-1, E-selectin, vWF, and uPA), GEM (IL-1α, IL-6, PECAM-1, ICAM-1, E-selectin, and vWF), and IFN-β1a (PECAM-1, ICAM-1, PAI-1, and uPA). Each drug induces MAC deposits on HMEC-1s and singular endothelial activation profiles, potentially leading to thrombogenesis observed in DITMA.

## 1 Introduction

Thrombotic microangiopathy (TMA) is a syndrome that combines microangiopathic hemolytic anemia, thrombocytopenia, and microthrombi, leading to ischemic tissue injury. It can affect various organs, such as the brain and the kidneys, among which the renal glomerular vessels appear particularly prone to endothelial damage and thrombus formation, leading to acute kidney injury (Schaefer et al., 2018).

TMA classifications are continuously evolving. Categories may include thrombotic thrombocytopenic purpura (TTP) due to a decreased disintegrin and metalloproteinase with thrombospondin-1 motifs 13th member (ADAMTS13) activity, atypical hemolytic uremic syndrome (aHUS) induced by alternative pathway dysregulation of the complement system, typical hemolytic uremic syndrome (tHUS) provoked by Shigatoxin-producing bacteria (classically *Escherichia coli*), and TMAs associated with specific conditions such as infections, pregnancy, medications or recreational drug use, transplantation, cancer, autoimmune diseases, and malignant hypertension (Fakhouri et al., 2017).

Endothelial cells (ECs) are key effectors of important physiological processes, including angiogenesis, vascular tone and permeability, regulation of smooth muscle cell proliferation, metabolism, inflammation, leukocyte trafficking, hemostasis, and coagulation (Jourde-Chiche et al., 2019). Optimal blood fluidity is ensured by healthy ECs expressing anticoagulant and antithrombotic properties due to the expression of various factors (tissue factor inhibitors, thrombomodulin, nitric oxide, and prostacyclin) (Jourde-Chiche et al., 2019; Neubauer et al., 2021). Triggers such as infections or inflammation may stress ECs and induce pro-inflammatory, pro-coagulant, or anti-fibrinolytic pathogenic activation profiles, potentially contributing to TMA (Jourde-Chiche et al., 2019). Growing evidence suggests that endothelial injury is a common underlying mechanism among various TMAs. Proposed endothelial damage mechanisms include alterations in ADAMTS13 activity, deregulation of the vascular endothelial growth factor (VEGF) and nuclear factor kappa light chain of B cell (NF-κB) pathway (Goldberg et al., 2010; Mathew et al., 2015; Blasco et al., 2022), and overactivation of the alternative pathway of the complement system (Kerr and Richards, 2012; Rotz et al., 2017). Drug-induced TMAs (DITMAs) account for more than 10% of all TMAs (Mazzierli et al., 2023) and 20%–30% of secondary TMAs (Allinovi et al., 2024). The mechanism of endothelial damage induced by different drugs is heterogeneous; however, DITMAs may occur by direct dose-dependent drug toxicity or immune-mediated endothelial damage (Al-Nouri et al., 2015; Chatzikonstantinou et al., 2020). Among the drugs with the strongest causative association with TMA, ciclosporin A (CsA), gemcitabine (GEM), and interferon β1a (IFN-β1a) have been reported in the literature (Sarkodee-Adoo et al., 2003; Humphreys et al., 2004; Kundra and Wang, 2017).

CsA, a calcineurin inhibitor, is a widely prescribed immunosuppressant for allograft rejection prevention and autoimmune disease treatment (Dunn et al., 2001). Although its clinical association with TMA has been previously highlighted (Al-Nouri et al., 2015), transplant-associated TMA frequently overlaps with CsA-induced TMA due to a shared clinical frame (Sabulski and Jodele, 2023).

GEM, a member of the deoxycytidine family of nucleoside analogs often used in treatment regimens of various solid tumors, induces cancerous cell-cycle arrest and apoptosis by insertion in the cell’s deoxyribonucleic acid during replication (Mosconi et al., 1997; Faivre et al., 1999; Mini et al., 2006). Cohort studies and case reports have confirmed its strong association with TMA, with an estimated incidence of 0.31%–1.4% in treated patients (Humphreys et al., 2004; Flombaum et al., 1999; Daviet et al., 2018; Burns et al., 2020). As with CsA, a clear diagnosis of GEM-induced TMA is often blurred in clinical practice by the overlapping presence of cancer, which may also induce TMA.

IFN-β1a, a pleiotropic cytokine approved for the treatment of relapsing–remitting multiple sclerosis, has multiple immunomodulatory properties attributed to yet partially unveiled mechanisms that seem mediated by transcriptional factors and subsequent gene regulations (Jakimovski et al., 2018). Its beneficial effect appears related to the downregulation of class II major histocompatibility complex expression on antigen-presenting cells, induction of interleukin (IL)-10 production by T cells, a cellular shift to anti-inflammatory T helper (Th)-2 cells, inhibition of T-cell migration by metalloproteases, and adhesion molecule blockade (Jakimovski et al., 2018). IFN-β1a also inhibits several pro-inflammatory cytokines, such as IL-6, IL-1β, tumor necrosis factor-alpha (TNF-α), and IFN-γ (Kieseier, 2011). Interestingly, IFN-β1a has been associated with TMA and poor renal outcomes (Allinovi et al., 2024; Kavanagh et al., 2016; Jia et al., 2018; Taghavi et al., 2022).

Most studies are based on clinical settings in patient cohorts to determine the relationship between drug exposure and TMA occurrence, although steps leading to DITMA, which seem dependent on drug type, remain unclear at the cellular level. Moreover, implications of the complement system in DITMA have been partially documented in clinical cases (i.e., with carfilzomib and IFN-β1a) (Taghavi et al., 2022; Allinovi et al., 2016; Bhutani et al., 2020; Aklilu and Shirali, 2023), but only a few *in vitro* studies have explored its underlying biological patterns (Lappin et al., 1992; Hunt et al., 2017; Blasco et al., 2020). Decreased C3 serum levels appear linked to increased complement protein deposits in kidney tissues, thus serving as a major marker of complement system activation and a useful predictive tool for complement inhibition therapy response (Mazzierli et al., 2023). Moreover, better renal outcomes in some patient series treated with eculizumab suggest a significant pathophysiological input of the complement system in DITMAs (Allinovi et al., 2024; Allinovi et al., 2016).

In an *in vitro* study comparing CsA, GEM, and IFN-β1a, often clinically implicated in DITMAs, we explored key features of endothelial pathogenic activation, such as inflammation (IL-1α and IL-6), leukocyte trafficking (platelet EC adhesion molecule-1 [PECAM-1], intercellular adhesion molecule-1 [ICAM-1], and E-selectin), hemostasis, and coagulation (plasminogen activator inhibitor-1 [PAI-1], urokinase plasminogen activator [uPA], and von Willebrand factor [vWF]), in microvascular ECs. Since morphological, physiological, phenotypical, and behavioral differences have been described between micro- and macrovascular EC lineages (Cines et al., 1998; Shi and Vanhoutte, 2017; Torramade-Moix et al., 2020), we experimented with human microvascular endothelial cell line-1 (HMEC-1) to benefit from their microvascular-size features and approach the capillary anomalies encountered in clinical studies involving DITMA (Mazzierli et al., 2023). We also sought to document complement activity in microvascular ECs during toxic aggression, to add input to the few *in vitro* studies that explored complement system activation in DITMA (Lappin et al., 1992; Hunt et al., 2017; Blasco et al., 2020).

## 2 Materials and methods

### 2.1 Cell culture

Immortalized HMEC-1s (American Type Culture Collection, Virginia, United States, ATCC, Cat# CRL-3243, RRID:CVCL_0307) were cultured between passages 5 and 12 in MCDB 131 medium (Gibco™, Thermo Fisher Scientific®, Belgium) supplemented with 10 mM L-glutamine (PAA Laboratories GmbH, Thermo Fisher Scientific®, Pasching, Austria), 10 ng/mL endothelial growth factor (EGF, Sigma-Aldrich® Merck KGaA®, Darmstadt, Germany), 1 μg/mL hydrocortisone (Gibco™, Thermo Fisher Scientific®, Belgium), 10% fetal bovine serum (FBS, Sigma-Aldrich® Merck KGaA®, Darmstadt, Germany), and 1% penicillin/streptomycin (Gibco™, Thermo Fisher Scientific®, Belgium) at 37 °C with 5% CO_2_ and saturated humidity. For experiments involving IL-1α and IL-6, hydrocortisone was not added to the medium, considering its anti-inflammatory properties.

Throughout our study, each assay was performed at least three times with two replicates each. HMEC-1s were exposed to 10% normal human serum (NHS), a human serum type AB pooled from male donors (A&E Scientific©, Belgian distributor of Capricorn Scientific GmbH®, Germany), diluted in MCDB 131 medium (1:10), as a source of complement system proteins (Brunn et al., 2006), CsA, GEM, or IFN-β1a. In each experiment, we studied HMEC-1 activation marker expressions in four treatment groups: controls (MCDB 131 medium), NHS (MCDB 131 medium + 10% NHS), drug (MCDB 131 medium + CsA, GEM, or IFN-β1a), and drug + NHS (MCDB 131 medium + 10% NHS + CsA, GEM, or IFN-β1a).

### 2.2 Cell viability

The respective cytotoxicity of CsA, GEM (Sigma-Aldrich® Merck KGaA®, Darmstadt, Germany), and IFN-β1a (Gibco™, Thermo Fisher Scientific®, Belgium) in HMEC-1 was evaluated using a resazurin reduction assay, which is based on the reduction of an indicator dye, resazurin, into the highly fluorescent resorufin by viable cells. Cells were treated with test compounds (CsA, GEM, or IFN-β1a) in 96-well plates (CELLSTAR®, Belgium). After 24 h, 48 h, and 72 h, cells were washed twice with phosphate-buffered saline (PBS, Sigma-Aldrich® Merck KGaA®, Darmstadt, Germany) and incubated with 0.44 mM resazurin solution (Sigma-Aldrich® Merck KGaA®, Darmstadt, Germany) at 37 °C for 2 h. The absorbance was measured at 540 and 620 nm wavelengths using an iEMS Reader MF spectrophotometer (Thermo Labsystems, Breda, Netherlands). Each assay was performed at least three times with two replicates per assay. Concentrations below IC_50_, allowing an acceptable cellular viability of 75% (IC_75_) with shorter incubation times (24 h), were selected to minimize time- and dose-related drug toxicity in further experiments.

### 2.3 Immunofluorescence (C5b-9 and vWF)

HMEC-1 cells were plated on cell culture slides (Greiner-Bio-One, Germany) and exposed to CsA (10 µM), GEM (10 nM), or IFN (1000 U/mL) for 24 h. Then, 10% NHS was added for 30 min at 37 °C and 5% CO_2_ in the NHS and drug + NHS wells. Signs of complement activation were assessed solely by demonstrating the presence of the complement cascade end-product, C5b-9 (membrane attack complex, MAC), deposited on HMEC-1s, treated as previously described.

After treatment, cells were washed in PBS and fixed in 4% paraformaldehyde (Klinipath, Netherlands) for 20 min at room temperature. Then, the cells were blocked with 2% bovine serum albumin (BSA, Sigma-Aldrich® Merck KGaA®, Darmstadt, Germany) for 1 h at room temperature. Cells were then incubated overnight at 4 °C in a humidified chamber with rabbit polyclonal anti-human C5b-9 (1:100; Sigma-Aldrich® Merck KGaA®, Darmstadt, Germany, Bioss, Cat# bs-2673R, RRID: AB_10855202), followed by a goat anti-rabbit IgG Alexa 488-labeled antibody (1:100; Abcam, Cambridge, United Kingdom, Abcam, Cat# ab150089, RRID:AB_2755130) for 30 min at 37 °C, or with rabbit polyclonal anti-human vWF (1:100; Santa Cruz Biotechnology, Dallas, Texas, United States, Santa Cruz Biotechnology, Cat# sc-14014, RRID: AB_2241707), followed by a goat anti-rabbit IgG Alexa 488-labeled antibody (1:100; Abcam, Cambridge, United Kingdom, Abcam, Cat# ab150089, RRID: AB_2755130) for 30 min at 37 °C. Coverslips were mounted with ProLong Gold Antifade reagent (Invitrogen, Thermo Fisher Scientific®, United States) containing 4′,6-diamidino-2-phenylindole (DAPI) to stain the nuclei. Imaging was obtained using a microscope (Carl Zeiss, Oberkochen, Germany) equipped with appropriate emission filters for rhodamine (Cypac, Brussels, Belgium) and captured using DeltaPix Viewer software (DeltaPix©, Denmark). Negative controls were obtained by omission of antibody 1, resulting in a black image (negative control), with DAPI staining of nuclei ensuring the presence of cells in the controls. The images were then uploaded to a computer and analyzed using NIH ImageJ software (ImageJ (RRID: SCR_003070)), which calculates averages of immunolabeled areas in pixels per field, calibrated on average DAPI-immunolabeled nuclei in pixels per field, starting from 10 fields per filter per well. Original images were converted to 8-bit format prior to binarization using ImageJ’s *Make Binary* auto-thresholding function. Binary images were generated to delineate regions of interest (ROIs) for quantitative analysis. A median filter was applied to reduce noise and normalize staining intensity distributions to median values. ROI areas were computed via the *Analyze Particles* function.

### 2.4 Measurement of intracellular IL-1α and IL-6

The intracellular IL-1α and IL-6 concentrations in HMEC-1 cells were determined by flow cytometry. In brief, following 24 h of treatment with CsA, GEM, or IFN-β1a in 12-well plates, the supernatants were removed and replaced with MCDB-131 and drugs, and the cells were incubated with 10% NHS at 37 °C and 5% CO2 for 30 min. Cells were harvested, centrifuged at 500 *g*, and fixed in Cytofix/Cytoperm solution (BD Biosciences, Erembodegem, Belgium) for 20 min at 4 °C. The suspension was washed and incubated with monoclonal antibody solutions of anti-human IL-1-FITC (Invitrogen, eBioscience™, Carlsbad, CA, United States, BD Biosciences, Cat# 551223, RRID: AB_394101) and anti-human IL-6-APC (Invitrogen, eBioscience™, Carlsbad, CA, United States, BD Biosciences, Cat# 554544, RRID: AB_395468) for 30 min in the dark at 4 °C. Afterward, cells were rinsed and analyzed using flow cytometry (FACS Canto II, BD Biosciences, San Jose, CA, United States). In every treatment condition (controls, NHS, drug, and drug + NHS), the mean immunofluorescence measured by flow cytometry was noted for each marker of interest (IL-1α, IL-6, E-selectin, ICAM-1, and PECAM-1). The means of the controls were calculated, and the other conditions (NHS, drug, and drug + NHS) were converted into percentages of the control means. The means of each condition percentage (%NHS, %Drug, and %Drug + NHS) were then integrated in GraphPad software [GraphPad Prism (RRID: SCR_002798)]; a one-way analysis of variance (ANOVA) and a Bonferroni post-test were performed to provide the level of significance for each treatment type.

### 2.5 Expression of adhesion molecules on the surface of HMEC-1 cells

Flow cytometry was used to assess the expression of E-selectin, ICAM-1, and PECAM-1 on HMEC-1s treated with CsA, GEM, and IFN-β1a. In brief, following 24 h of treatment with CsA, GEM, or IFN-β1a in 12-well plates, the supernatants were removed and replaced with MCDB-131 medium and drugs, and the cells were incubated with 10% NHS at 37 °C and 5% CO_2_ for 30 min. The suspension was washed and incubated with monoclonal antibody solutions of anti-human E-selectin-FITC (Invitrogen, eBioscience™, Carlsbad, CA, United States, Thermo Fisher Scientific, Cat# MA5-46666, RRID: AB_2937738), anti-human ICAM-1-APC (Invitrogen, eBioscience™, Carlsbad, CA, United States, Thermo Fisher Scientific, Cat# 17-0549–41, RRID: AB_10718240), and anti-human PECAM-1-FITC (Invitrogen, eBioscience™, Carlsbad, CA, United States, Thermo Fisher Scientific, Cat# 11-0319–41, RRID: AB_2043836) for 30 min in the dark at 4 °C. Afterward, cells were rinsed and analyzed via flow cytometry (FACS Canto II, BD Biosciences, San Jose, CA, United States). Immunofluorescence recordings were then calculated as percentages of control means, and the values were integrated in GraphPad software [GraphPad Prism (RRID: SCR_002798)], as previously described.

### 2.6 Secretion of fibrinolytic and anti-fibrinolytic molecules in the supernatants of HMEC-1s

PAI-1 (R and D Systems, Cat# DY9387-05, RRID: AB_3083723) and uPA (R and D Systems, Cat# BAF1310, RRID: AB_2165329) protein levels in the supernatants of cultured HMEC-1s were measured using commercially available sandwich enzyme-linked immunosorbent assay (ELISA) kits (R&D, Minneapolis, United States), according to the manufacturer’s instructions. In brief, these ELISA kits comprise pre-coated micro-test wells with anti-human PAI-1 or anti-human uPA antibodies, which bind to their respective antigens if present in the supernatants. After incubation, secondary biotinylated antibodies directed against the bound antibodies to PAI-1 or uPA molecules were added to the supernatants. The antibody–enzyme detection complex was completed by adding 100 μL of streptavidin horseradish peroxidase (HRP, Sigma-Aldrich® Merck KGaA®, Darmstadt, Germany), and the supernatants were supplemented with 90 μL of tetramethylbenzidine (TMB, Sigma-Aldrich® Merck KGaA®, Darmstadt, Germany) substrate, forming a blue-colored solution. The final absorbance was analyzed at 450 nm using an iEMS Reader MF spectrophotometer (Thermo Labsystems, Breda, Netherlands). PAI-1 and uPA levels were then determined according to a calibration curve generated from a set of PAI-1 and uPA standards provided by the manufacturer.

### 2.7 Statistical analysis

All data were expressed as the means ± standard deviation (mean ± SD). Differences between cell culture conditions were evaluated by ANOVA, followed by a *post hoc* analysis using Bonferroni’s multiple comparison test in Prism software 5 [GraphPad Software, Inc., San Diego, CA, United States, GraphPad Prism (RRID: SCR_002798)]. The mean values and SDs were calculated from at least three independent experiments with two replicates each. The *p*-value was considered significant when below 0.05.

## 3 Results

### 3.1 Effect of drugs on cell viability

**FIGURE 1:**
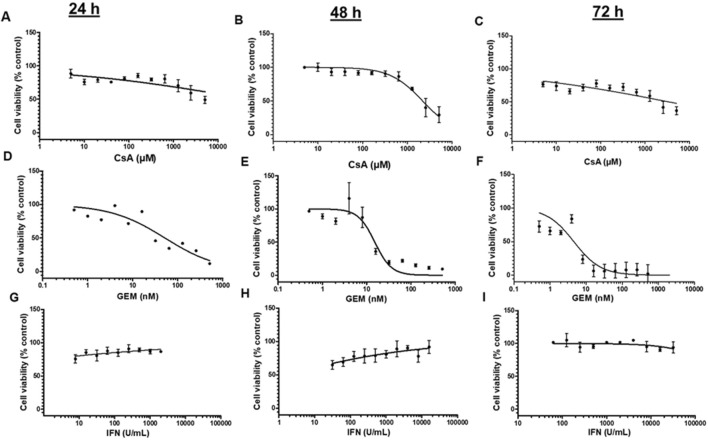
Determination of the dose–response curves using the resazurin test in HMEC-1s following a 24-, 48-, or 72-h exposure to CsA **(A–C)**, GEM **(D–F)**, and IFN-β1a [IFN; **(G–I)**], respectively. Values are presented as the means ± SD from three independent experiments with two replicates each.

Cytotoxic concentration ranges of CsA, GEM, and IFN-β1a were determined in HMEC-1s using the resazurin test. Doses that significantly reduced HMEC-1 viability after 24 h, 48 h, and 72 h compared with the control group were determined (Figure 1). GEM appeared most toxic at 48 h and 72 h by significantly reducing cellular viability in a dose-dependent manner (Figures 1 E,F). At 72 h, GEM’s IC_50_ (4.546 nM ± 1) was lower than that of CsA (3.286 µM ± 2) and IFN-β1a, the latter showing no cytotoxicity even after 72 h at high concentrations. Concentrations allowing an acceptable cellular viability of 75% below IC_50_ with shorter incubation times (24 h) were selected for further experiments: 10 µM CsA (Figure 1A), 10 nM GEM (Figure 1D), and 1000 U/mL IFN-β1a (Figure 1G).

### 3.2 C5b-9 deposition on HMEC-1s

**FIGURE 2:**
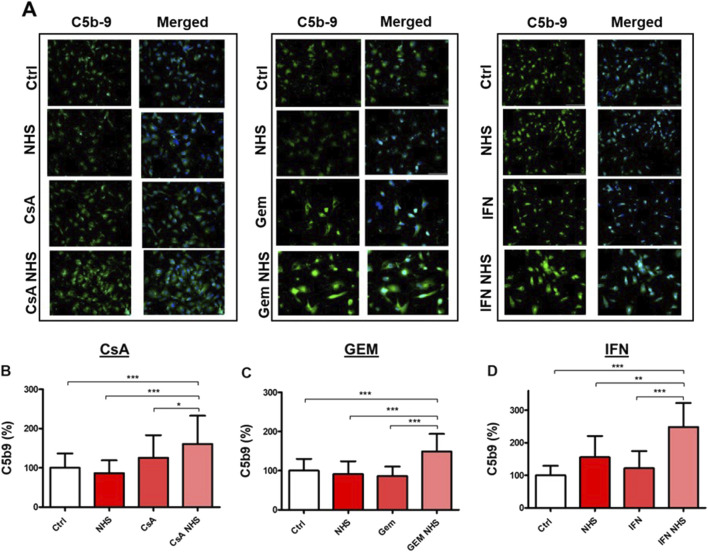
Determination by immunofluorescence of drug-induced C5b-9 deposition on HMEC-1s following a 24-hour exposure to CsA (10 μM), GEM (10nM), and IFN-β1a (1000 U/mL) (IFN), respectively. **(A)** Representative microscopic findings of C5b-9 deposition (green staining) on HMEC-1s (blue DAPI–stained nuclei). **(B–D)** Bar diagrams representing quantification of C5b-9 deposits expressed as fold increase of the covered surface compared to control (Ctrl). Values are presented as the means ± SD from three independent experiments with two replicates each. * *p* < 0.05, ** *p* < 0.01, *** *p* < 0.001.

C5b-9 deposition was measured using immunofluorescence (Figure 2A). No significant C5b-9 deposits were found with NHS alone or following CsA, GEM, or IFN-β1a treatment alone (Figures 2B–D). However, C5b-9 deposits significantly increased when HMEC-1s were exposed to each drug individually with NHS (Figures 2B–D) compared to controls (CsA, *p* < 0.001; GEM, *p* < 0.001; IFN-β1a, *p* < 0.001), NHS alone (CsA, *p* < 0.001; GEM, *p* < 0.001; or IFN-β1a, *p* < 0.01), or each drug alone (CsA, *p* < 0.05; GEM, *p* < 0.001; or IFN-β1a, *p* < 0.001).

### 3.3 Interleukin expression in HMEC-1s

**FIGURE 3:**
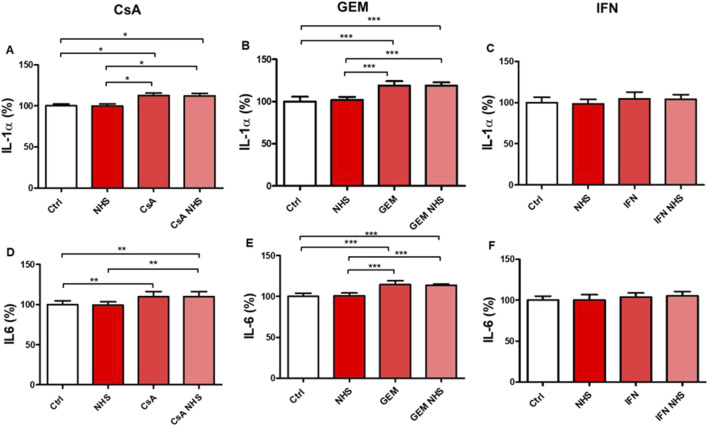
Determination by flow cytometry of IL-1α and IL-6 expression in HMEC-1s following a 24-hour exposure to CsA **(A, D)**, GEM **(B, E)**, or IFN-β1a (IFN; **C, F**), respectively. Values are presented as the means ± SD from three independent experiments with two replicates each. * *p* < 0.05, ** *p* < 0.01, *** *p* < 0.001.

Pro-inflammatory markers IL-1α and IL-6 were measured by flow cytometry in HMEC-1s exposed to CsA, GEM, or IFN-β1a. Increased expression of IL-1α and IL-6 appeared in HMEC-1s treated with CsA and GEM compared with controls, both in the presence and absence of NHS (Figures 3A,B,D,E).

For CsA, IL-1α levels (Figure 2A) were significantly increased in CsA with NHS compared with controls (*p* < 0.05) or NHS alone (*p* < 0.05) and CsA alone compared with controls (*p* < 0.05) or NHS alone (*p* < 0.05). Similarly, IL-6 levels (Figure 2D) were significantly increased in CsA with NHS compared with controls (*p* < 0.01) or NHS (*p* < 0.01), and CsA alone compared with controls (*p* < 0.01).

With GEM, IL-1α levels (Figure 2B) were significantly increased in treatment groups GEM with NHS compared with controls (*p* < 0.001) or NHS (*p* < 0.001) and GEM alone compared with controls (*p* < 0.001) and NHS alone (*p* < 0.001). IL-6 levels (Figure 2E) were also significantly increased in GEM with NHS compared with controls (*p* < 0.001) or NHS (*p* < 0.001), and GEM alone compared with controls (*p* < 0.001) or NHS (*p* < 0.001).

IL-1α and IL-6 production by HMEC-1s was unaffected by exposure to IFN-β1a (Figures 3C,F).

### 3.4 Expression of adhesion molecules in HMEC-1s exposed to CsA, GEM, or IFN-β1a

**FIGURE 4:**
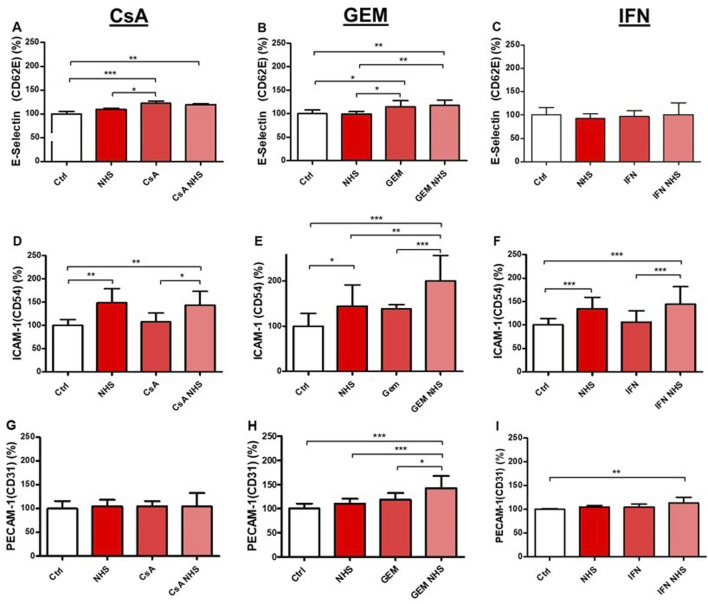
Determination by flow cytometry of E-selectin (CD62E), ICAM-1 (CD54), and PECAM-1 (CD31) expression in HMEC-1s following a 24-hour exposure to CsA **(A, D, G)**, GEM **(B, E, H)** or IFN-β1a (IFN; **C, F, I**), respectively. Values are presented as the means ± SD from three independent experiments with two replicates each. * *p* < 0.05, ** *p* < 0.01, *** *p* < 0.001.

HMEC-1 expression levels of E-selectin, ICAM-1, and PECAM-1 were investigated by flow cytometry. With CsA (Figure 4A), E-selectin levels increased in treatment groups CsA with NHS compared with controls (*p* < 0.01) and in CsA alone compared with controls (*p* < 0.001) or NHS (*p* < 0.05). GEM (Figure 4B) increased E-selectin expression in treatment groups GEM with NHS compared with controls (*p* < 0.01) or NHS (*p* < 0.01) and in GEM alone compared with controls (*p* < 0.05) or NHS (*p* < 0.05). IFN-β1a exposure did not affect E-selectin levels in HMEC-1s (Figure 4C).

Increased ICAM-1 expression was observed with NHS alone and, logically, also when each drug was combined with NHS (Figures 4D,E,F). CsA with NHS (Figure 4D) significantly increased ICAM-1 levels compared with controls (*p* < 0.01) or CsA alone (*p* < 0.05). GEM with NHS significantly increased ICAM-1 levels (Figure 4E) compared with controls (*p* < 0.001), NHS (*p* < 0.01), or GEM alone (*p* < 0.001). IFN-β1a with NHS (Figure 4F) significantly increased ICAM-1 expression compared with controls (*p* < 0.001) or IFN-β1a alone (*p* < 0.001).

In HMEC-1s, PECAM-1 expression was unaffected by CsA (Figure 4G). Exposure to GEM with NHS (Figure 4H) significantly increased PECAM-1 levels compared with controls (*p* < 0.001), NHS alone (*p* < 0.001), or GEM alone (*p* < 0.05). IFN-β1a with NHS (Figure 4I) significantly increased PECAM-1 expression compared with controls (*p* < 0.01).

### 3.5 Expression of PAI-1 by HMEC-1s

**FIGURE 5:**
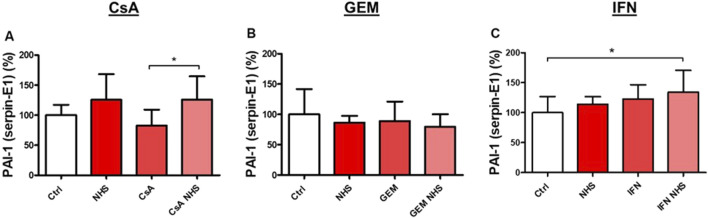
Determination by ELISA of PAI-1 (serpin-E1) levels in the supernatants of HMEC-1s following a 24-hour exposure to CsA **(A)**, GEM **(B)**, or IFN-β1a (IFN) **(C)** . Values are presented as the means ± SD from three independent experiments with two replicates each. * *p* < 0.05, ** *p* < 0.01, *** *p* < 0.001.

PAI-1 levels were measured in the supernatants of HMEC-1s using ELISA. Exposure to CsA with NHS (Figure 5A) significantly increased PAI-1 expression compared to CsA alone (*p* < 0.05). Treatment with GEM did not affect PAI-1 levels in HMEC-1s (Figure 5B). IFN-β1a with NHS (Figure 5C) increased PAI-1 expression compared to controls (*p* < 0.05).

### 3.6 Expression of uPA by HMEC-1s

**FIGURE 6:**
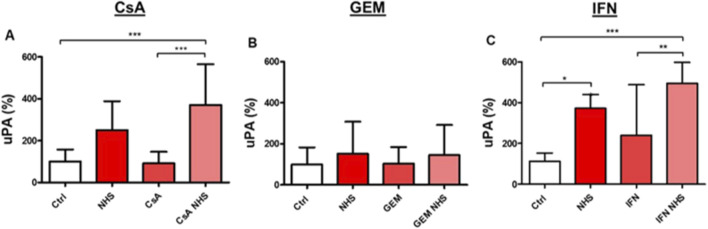
Determination by ELISA of urokinase Plasminogen Activator (uPA) levels in the supernatants of HMEC-1s following a 24-hour exposure to CsA **(A)**, GEM **(B)**, or IFN-β1a (IFN) **(C)**, respectively. Values are presented as the means ± SD from three independent experiments with two replicates each. * *p* < 0.05, ** *p* < 0.01, *** *p*<0.001.

Levels of the pro-thrombotic enzyme uPA were measured using ELISA in the supernatants of HMEC-1s (Figure 6). CsA exposure (Figure 6A) significantly increased uPA secretion compared with controls (*p* < 0.001) or CsA alone (*p* < 0.001). GEM did not affect uPA secretion in HMEC-1s (Figure 6B). As with CsA, IFN-β1a (Figure 6C) induced significant uPA secretion compared with controls (*p* < 0.001) or IFN-β1a alone (*p* < 0.01). In our assay, NHS alone on HMEC-1s appeared to be associated with variable supernatant concentrations of uPA, which may be significant (*p* < 0.05) compared with controls (Figure 6C).

### 3.7 Expression of membranous vWF by HMEC-1s

**FIGURE 7:**
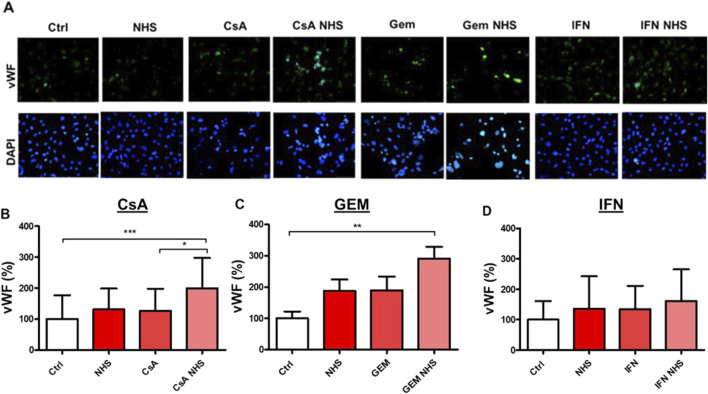
Determination by immunofluorescence of membranous vWF expression in HMEC-1s after a 24-hour exposure to CsA, GEM, or IFN-β1a (IFN), respectively. **(A)** Representative microscopic findings of membranous vWF expression (green staining) on HMEC-1s (blue DAPI–stained nuclei). (**B–D**) Bar diagrams representing membranous vWF expression quantified as a fold-increase of the covered surface compared to controls. Values are presented as the means ± SD from three independent experiments with two replicates each. * *p* < 0.05, ** *p* < 0.01, *** *p* < 0.001.

Membranous vWF expression by HMEC-1s was measured by immunofluorescence (Figure 7A). CsA with NHS (Figure 7B) significantly increased membranous vWF expression compared with controls (*p* < 0.001) or CsA alone (*p* < 0.05). GEM with NHS (Figure 7C) significantly increased membranous vWF expression compared with controls (*p* < 0.01). HMEC-1 membranous vWF levels were not affected by IFN-β1a treatment (Figure 7D).

### 3.8 Summary of the results

**FIGURE 8:**
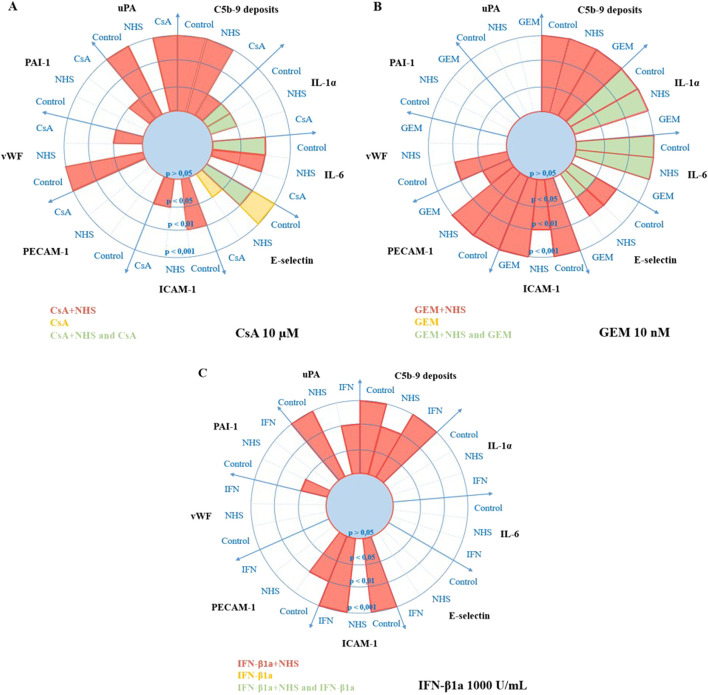
Radar chart representations of HMEC-1 activation marker expressions when exposed to ciclosporin A (CsA) **(A)**, gemcitabine (GEM) **(B)**, or interferon-β1a (IFN) **(C)** for 24 h in four treatment conditions (control [MCDB 131], NHS, drug, and drug + NHS). Statistically significant expression levels compared to other treatment groups are colored in red for drug + NHS, yellow for drug alone, and green for overlapping drug and drug + NHS levels. Values are expressed as the means ± SD from three independent experiments with two replicates each, in a four-level axis of statistical significance (*p* > 0.05, *p* < 0.05, *p* < 0.01, and *p* < 0.001). Data obtained from immunofluorescence (for C5b-9 and vWF), flow cytometry (for IL-1α, IL-6, E-selectin, ICAM-1, and PECAM-1), or enzyme-linked immunosorbent assay (ELISA, for PAI-1, and uPA). HMEC-1, human microvascular endothelial cell line-1; NHS, normal human serum; C5b-9, complement membrane attack complex; vWF, von Willebrand factor; IL, interleukin; ICAM-1, intercellular adhesion molecule-1; PECAM-1, platelet endothelial cell adhesion molecule-1; PAI-1, plasminogen activator inhibitor-1; uPA, urokinase plasminogen activator.

The results obtained from immunofluorescence, flow cytometry, and ELISA on HMEC-1 activation markers when exposed to NHS after a 24-h treatment with IC_75_ CsA, GEM, and IFN-β1a are summarized in Figure 8.

Each drug with NHS significantly increased C5b-9 deposits on HMEC-1s compared with controls, NHS, and the drug alone. NHS alone on HMEC-1s consistently increased ICAM-1 levels (*p* < 0.05 to *p* < 0.001) and variably increased supernatant concentrations of uPA, which may be significant (*p* < 0.05) compared with controls (Figure 6C; Figure 8).

CsA with NHS increased membranous expression of vWF (compared to controls [*p* < 0.001], and CsA [*p* < 0.05]), E-selectin (compared to controls [*p* < 0.01]), and ICAM-1 (compared to controls [*p* < 0.01], and CsA [*p* < 0.05]). CsA with NHS also increased intracellular levels of IL-1α (compared to controls [*p* < 0.05] or NHS [*p* < 0.05]) and IL-6 (compared to controls [*p* < 0.01] or NHS [*p* < 0.01]), as well as supernatant levels of PAI-1 (compared to CsA [*p* < 0.05]) and uPA (compared to controls [*p* < 0.001], or CsA [*p* < 0.001]). CsA alone significantly increased the expressions of IL-1α (compared to controls [*p* < 0.05] or NHS [*p* < 0.05]), IL-6 (compared to controls [*p* < 0.01]), and E-selectin (compared to controls [*p* < 0.001] or NHS [*p* < 0.05]).

GEM with NHS increased membranous expression of vWF (compared to controls [*p* < 0.01]), E-selectin (compared to controls [*p* < 0.01], or NHS [*p* < 0.01]), ICAM-1 (compared to controls [*p* < 0.001], NHS [*p* < 0.01], or GEM [*p* < 0.001]), and PECAM-1 (compared to controls [*p* < 0.001], NHS [*p* < 0.001], or GEM [*p* < 0.05]). GEM with NHS also increased intracellular levels of IL-1α (compared to controls [*p* < 0.001] or NHS [*p* < 0.001]) and IL-6 (compared to controls [*p* < 0.001] or NHS [*p* < 0.001]). GEM alone significantly increased the expression of IL-1α (compared to controls [*p* < 0.001], or NHS [*p* < 0.001]), IL-6 (compared to controls [*p* < 0.001], or NHS [*p* < 0.001]), and E-selectin (compared to controls [*p* < 0.05], or NHS [*p* < 0.05]).

IFN-β1a with NHS increased membranous expressions of ICAM-1 (compared to controls [*p* < 0.001] or IFN-β1a [*p* < 0.001]) and PECAM-1 (compared to controls [*p* < 0.01]). IFN-β1a with NHS also increased supernatant levels of PAI-1 (compared to controls [*p* < 0.05]) and uPA (compared to controls [*p* < 0.001] or IFN-β1a [*p* < 0.01]). IFN-β1a alone did not affect the expression of the markers we studied.

## 4 Discussion

In this experimental work, we aimed to evaluate the effect of three different drugs implicated in DITMAs on microvascular endothelial cells, HMEC-1s, which constitutively express PAI-1, tissue-type plasminogen activator (tPA), vWF, and thrombomodulin (Ribeiro et al., 1995). These cells proved to be a reliable EC line for *ex vivo* demonstration of complement activity in atypical hemolytic uremic syndrome (aHUS) patients (Noris et al., 2014). Since aHUS typically involves microvascular ECs with TMA features, particularly in the kidneys (Nor et al., 2014), we chose to experiment with HMEC-1s over other primary EC lines such as human umbilical vein endothelial cells (HUVECs). As in previous publications (Xie et al., 2022; Mulfaul and Doyle, 2021; Jeon et al., 2014), we experimented with a 10% NHS concentration to approach the globulin protein fraction of complement compounds in human plasma (Bardhan and Kaushik, 2022).

Cytotoxicity and cell growth were determined using the resazurin assay, which detects the presence of live cells with functional mitochondria. GEM, as expected, reduced viability since this anti-proliferative agent engages in the S phase of the cell cycle in actively dividing cells (Laquente et al., 2008), including ECs (van Hell et al., 2017). At the selected concentrations in our experiments, neither CsA nor IFN-β1a appeared significantly cytotoxic. Other authors have reported different viabilities depending on the concentration of CsA used and the EC type studied (Rafiee et al., 2002; Bouvier et al., 2008; Carmona et al., 2013). For instance, Jia et al. (2018) reported a dose-dependent viability decrease in macrovascular HUVECs treated with IFN-β1a. This suggests that different EC types could express various IFN receptor compositions and alternative intracellular pathway activations, leading to divergent toxic effects (Jia et al., 2018). Because both CsA and IFN-β1a appeared mildly or non-cytotoxic in our experiments, other underlying endothelial activation mechanisms potentially involved in DITMA were investigated.

Measuring C5b-9 deposits on EC membranes has proven to be a useful assessment tool to explore the complement system’s overactivation (Galbusera et al., 2019; Palomo et al., 2019). Since NHS provided complement proteins, we examined endothelial responses in its presence and absence. When activated, complement proteins engage in a serine–protease cascade leading to the formation of the terminal complement complex (C5b-9), also known as the MAC (Noris and Remuzzi, 2013). A large body of evidence suggests that sublytic concentrations of MACs on EC membranes can induce the translocation of cytosolic NF-κB into the nucleus, leading to cytokine secretion involved in endothelial activation (Brunn et al., 2006; Kilgore et al., 1997). Our study showed that NHS with CsA, GEM, and IFN-β1a was associated with membranous C5b-9 deposits on microvascular HMEC-1s, implying that these three drugs may trigger the complement system and provoke sublytic MAC levels on these cells, potentially activating them.

We then observed that CsA and GEM significantly increased IL-1α and IL-6 expression independently of NHS, implying that signaling pathways other than the sublytic MAC deposits probably mediate them. Endothelial endoplasmic reticulum stress detected with CsA on human umbilical artery endothelial cells (HUAECs) (Bouvier et al., 2008) and reduced terminal sialic acid expression in the endothelial glycocalyx with GEM on HUVECs (Gunji et al., 2023) could both explain the increased production of inflammatory cytokines observed in our experiments. Cross-talk between inflammation and thrombogenesis has been previously demonstrated. Orbe et al. (1999) showed that IL-1α enhanced PAI activity and subsequent fibrinolysis inhibition in HUVECs. In addition, inflammatory mediators such as IL-6 can increase platelet responsiveness to thrombin, leading to a greater thrombogenic potential (Burstein, 1997; Esmon, 2004). Some studies even suggest that pro-inflammatory cytokines, including IL-1 and IL-6, could induce the expression of tissue factor (TF, coagulation factor III) on circulating monocytes and EC surfaces (Doshi and Marmur, 2002; Sungurlu et al., 2020). Moreover, other coagulation factors, such as fibrinogen and factor VIII, are promoted by IL-6 (Stouthard et al., 1996; Stirling et al., 1998). In our experiments on IFN-β1a, the IL-1α and IL-6 levels measured were low, both in the presence and absence of NHS. This suggests that IFN-β1a-induced sublytic MAC deposits on HMEC-1s do not affect the expression of these cytokines and that secretions of PAI-1 and uPA were not influenced by IL-1α and IL-6 under this drug. Although HMEC-1s exposed to CsA with NHS showed increased sublytic MAC deposition and elevated IL-1α, IL-6, PAI-1, uPA, and vWF expression, deciphering a causal relationship between cytokine expression, thrombogenesis, and the sublytic MACs observed in these ECs would require further experiments.

E-selectins are endothelial transmembrane glycoproteins responsible for low-affinity calcium-dependent ligations involved in intercellular recognition, growth inhibition, and diapedesis (Mittal et al., 2014). On activated ECs, E-selectins allow cellular attachment and rolling on their surface as a first step of the adhesion cascade (Imhof and Dunon, 1995; Brevetti et al., 2006). In our experiments, E-selectin expression increased in HMEC-1s exposed to CsA and GEM, with or without NHS, compared to controls. In intestinal microvascular ECs, Rafiee et al. (2002) suggested that CsA may exert multiple effects on vascular cells, resulting in increased leukocyte adhesion through inhibition of p38 mitogen-activated protein kinase (MAPK) and inducible nitric oxide synthetase (iNOS). Similarly, Mittal et al. (2014) demonstrated that GEM activated the NF-κB transduction pathway and transcription of endothelial E-selectin via oxidative stress, potentially explaining the increased GEM-induced E-selectin expression compared to controls found in our data. Conversely, E-selectin expression was unaffected by IFN-β1a exposure independently of NHS. This concords with a previous study on human brain-derived microvascular endothelial cells (HBMECs), reporting that cell adhesion phenomena were not influenced by IFN-β1a (Defazio et al., 2001).

ICAM-1 is a transmembrane protein related to immunoglobulins, liable for cell rolling arrest via high-affinity bonds to initiate cellular transmigration through the endothelium (Mittal et al., 2014). Our experiments on HMEC-1s indicated that ICAM-1 expression was unaffected by CsA, GEM, or IFN-β1a alone. Meanwhile, NHS alone increased ICAM-1, suggesting that components of NHS could favor ICAM-1 surface expression in HMEC-1s, as indicated by a previous study showing ICAM-1 upregulation via phosphorylation of ICAM-1 membrane tyrosine residues (Mittal et al., 2014). Only GEM with NHS significantly induced ICAM-1 expression compared to NHS alone, suggesting a specific role for this drug involving either complement activation, GEM-induced oxidative stress, or both. Within the limits of our experiments on HMEC-1s, a clear relationship between ICAM-1 expression and sublytic MAC deposits following drug exposure remains undetermined.

PECAM-1 is a transmembrane protein with high-affinity bonds to its ligands, which allows transmigration through the intercellular junctions and intervenes in various cell processes such as angiogenesis, intracellular signal transduction, vascular remodeling, and mechano-sensitivity (Mittal et al., 2014; Privratsky et al., 2010; Kitazume et al., 2014). Our results indicated that PECAM-1’s expression in HMEC-1s remained unchanged after treatment with CsA, GEM, or IFN-β1a alone. However, it increased when GEM or IFN-β1a was combined with NHS. Another study on HUVECs with higher GEM concentrations and prolonged exposure periods (48 h) showed decreased PECAM-1 expression, attributed to alterations in glycocalyx sialic acids, provoking PECAM-1 de-dimerization and internalization toward the nuclei (Gunji et al., 2023). In our study, IFN-β1a with NHS increased PECAM-1 expression compared with controls. Concordant with our results, Nelissen et al. (2002) found a PECAM-1 upregulation in HUVECs exposed to IFN-β1a, and Kraus et al. (2004) showed a direct *in vitro* stabilizing effect of IFN-β1a on cerebral blood–brain barrier ECs. Hypothetically, endothelial activation involving IFN-β1a-induced sublytic MAC deposits could activate the signal transducer and activator of transcription 3 (STAT3) pathway, known to promote PECAM-1 synthesis in an anti-inflammatory fashion (Privratsky et al., 2010). Although beyond the reach of our experiments, this IFN-β1a-related anti-inflammatory effect could explain the absence of IL-1α and IL-6 expressions we observed in HMEC-1s.

PAI-1, an inhibitor of tPA and uPA, is a known promoter of thrombus formation (Huber et al., 2001). In our experiments on HMEC-1s, CsA with NHS increased PAI-1 secretion and sublytic MAC deposits compared with controls. Given that the PAI-1 gene promoter seems inducible by NF-κB in HUVECs (Swiatkowska et al., 2005) and that sublytic MAC deposits are also known as NF-κB inducers (Brunn et al., 2006; Kilgore et al., 1997), it is possible that the pathway leading to increased PAI production in HMEC-1s exposed to CsA involves NF-κB activation and sublytic MAC deposits. Yet, PAI expression was unaffected by GEM in HMEC-1s, despite activating NF-κB (Mittal et al., 2014). Allegedly, GEM primarily induces TMA through a direct and cumulative cytotoxic effect on ECs, with an often long delay between the first dose and TMA occurrence in clinical practice (Daviet et al., 2018; Izzedine et al., 2006). Because its toxic effect on ECs, leading to platelet aggregation and complement activation, is dose- and time-dependent (Aklilu and Shirali, 2023), potential associations between PAI-1 secretion and GEM-induced sublytic MACs should be assessed at other concentrations and over longer treatment periods in future experimental studies. In HMEC-1 supernatants, we found that IFN-β1a with NHS significantly increased PAI-1 secretion compared with controls. These findings are similar to those of Jia et al. (2018), who demonstrated an upregulation of PAI-1 after IFN-β1a exposure in HUVECs, effectively disrupting the fibrinolysis system.

In the supernatants of HMEC-1s, increased uPA expression was observed after CsA and IFN-β1a treatment with NHS exposure compared with controls and the drug alone, similar to PAI-1. Both uPA and PAI-1 are upregulated through NF-κB activation (Swiatkowska et al., 2005), which also seems inducible by CsA in endothelial colony-forming cells (Meyer et al., 2021). Moreover, IFN-β1a exposure enhances PAI-1 but reduces uPA expression in HUVECs (Jia et al., 2018), while it increases both PAI-1 and uPA in HMEC-1s, probably due to behavioral differences depending on EC vessel-type origin and size. Moreover, GEM failed to induce uPA expression in HMEC-1s, despite activating NF-κB in ECs (Mittal et al., 2014). Because of its cell-cycle arrest properties, GEM probably limits uPA secretion via another pathway as uPA expression is low in resting ECs (Stepanova et al., 2016).

Our experiments revealed a significant increase in membranous vWF expression in HMEC-1s exposed to CsA and GEM with NHS compared with controls. These results may be associated with the activation of the complement system attributable to the C5b-9 deposits we demonstrated since binding of C5a to its endothelial receptor, C5R1, is known to provoke Weibel–Palade body exocytosis, thus exposing large vWF multimers in the bloodstream (Aiello et al., 2021). As previously demonstrated on HUVECs (Jia et al., 2018), IFN-β1a with NHS failed to induce significant vWF expression, regardless of sublytic MAC deposits on HMEC-1s. This observation suggests missing links in IFN-β1a-induced HMEC-1 exocytosis of vWF, requiring further experiments, which would also benefit from measuring intracellular and supernatant vWF levels to detect potential differences in its expression, whether bound or unbound to the endothelial microvascular membrane.

Although this *in vitro* study documents various significant levels of protein expression in HMEC-1s exposed to CsA, GEM, and IFN-β1a, several limitations require discussion. Each technique used to assess activation marker expressions has strengths and weaknesses, which could be compensated for by combining and comparing both flow cytometry and immunofluorescence assays in membranous adhesion marker expressions, for example, since the solubilization process required for flow cytometry experiments may influence their levels and membrane distributions. Examining the supernatants using ELISA kits would also inform on potential shedding differences due to specific technique processes. A two-dimensional HMEC-1 single-cell-type monolayer *in vitro* culture exposed to different drugs is a simplistic model of the endothelium *in vivo*, which insufficiently explores the complex pathophysiology clinically at stake in DITMA. Although TMA features may appear experimentally, questions regarding their pathological implications, interactions with the coagulation system, and platelet–immune–endothelial cell intercellular reactions remain unanswered using this model. In the vascular wall, smooth muscle and fibroblasts interplay with drug-exposed ECs, while blood flow, pressure, and shear stress, which are crucial in TMA, all certainly need to be studied in further experiments. Moreover, the effects of chronic drug exposure on the endothelium cannot be replicated *in vitro*, and HMEC-1s are immortalized microvascular cells whose activated molecular markers *in vitro* may differ from cultured primary EC lines and organ-specific ECs. Along these lines, although results may be statistically significant *in vitro*, they may actually represent side effects or satellite events of the underlying activation pathway at stake. Although they are highlighted experimentally, they may not be essential elements of the endothelial activation process *in vivo* and may, therefore, divert attention from the key reactions at play. Careful interpretation of data is required; conditions with slightly significant experimental differences may not be biologically or clinically significant, while negative results can be attributed to certain limits of the experimental technique used and still provide useful information in the search process of an activation pathway.

In summary, our results indicate a common feature in HMEC-1 cells exposed to each drug with NHS: sublytic C5b-9 membranous deposits and varying levels of increased activation markers. Although a *p*-value <0.05 was considered significant, true clinical involvement in an activation pathway may be greater in results with the highest statistical significance. Moreover, combined statistical significance in the treatment groups, drug alone and drug with NHS, compared to controls or NHS, probably suggests drug-related, rather than NHS-component, effects on activation marker expression. In the search for an activation pathway involving these markers and sublytic MAC deposits on HMEC-1s, the most statistically significant results in drugs with NHS treatments, especially when compared to all other treatment groups, are probably the best clues to evaluate in future experiments. In our study, CsA appeared moderately cytotoxic and inflammatory, was mildly involved in leukocyte trafficking, and showed greater anti-fibrinolytic properties. GEM was very cytotoxic and inflammatory, increased adhesion marker expression, and lacked anti-fibrinolytic properties. HMEC-1 exposure to IFN-β1a appeared to be the most different; it was neither cytotoxic nor inflammatory, and it moderately increased leukocyte trafficking markers and fibrinolytic protein secretion. The determination of a link between the sublytic MAC deposits and the activation marker expressions we observed would require other experiments, including statistically significant activation markers, specific inhibitors, C5b-9-positive and -negative controls, and calcium influx-positive and -negative controls.

## 5 Conclusion

CsA, GEM, and IFN-β1a exposures on HMEC-1s all trigger the complement system within NHS *in vitro*. Despite inducing similar sublytic MAC deposits, each drug influences different pro-inflammatory and pro-thrombotic activation profiles depending on EC type, potentially leading to the thrombogenesis observed in DITMA. The determination of a causal link between sublytic C5b-9 deposits and the particular HMEC-1 activations observed will be addressed in future studies as they may both result from and contribute to activation (Jokiranta, 2017). Transposition of *in vitro* observations into a clinical setting of DITMA would also benefit from different experimental techniques, models, and EC culture types to identify possible curative or preventive therapies more targeted than terminal complement cascade pathway inhibitors.

## Data Availability

The raw data supporting the conclusions of this article will be made available by the authors, without undue reservation.
