# Supplementary material for: In vitro exploration of drug-induced thrombotic microangiopathies: clues of diverse endothelial activation pathways respective to interferon-β1a, ciclosporin A, and gemcitabine exposure
Source: Front Pharmacol. 2026 Feb 2;16:1719192. doi: 10.3389/fphar.2025.1719192 (PMC12907546; doi:10.3389/fphar.2025.1719192)
Supplement: Supplementary file 1 [file Supplementaryfile1.docx]

**Supplementary material**

**Supplementary Figure 1.** Flow cytometry histograms of IL-1α and IL-6 expression in HMEC-1s following a 24-hour exposure to CsA, GEM, or IFN-β1a (IFN), respectively. Values are means ± SD from three independent experiments.

**Supplementary Figure 2.** Flow cytometry histograms of E-selectin (CD62E), ICAM-1 (CD54), and PECAM-1 (CD31) expression in HMEC-1s following a 24-hour exposure to CsA, GEM or IFN-β1a, respectively. Values are means ± SD from three independent experiments.
